# The relationship between self-compassion and mobile phone addiction in university students: a chain mediation model

**DOI:** 10.3389/fpsyt.2025.1603860

**Published:** 2025-06-17

**Authors:** Jingyu Qiang, Ying Jin, Yuqi Li, Cheng Xu

**Affiliations:** ^1^ School of Foreign Languages, Guangdong University of Petrochemical Technology, Maoming, Guangdong, China; ^2^ School of Psychology and Cognitive Science, East China Normal University, Shanghai, China

**Keywords:** self-compassion, negative attention bias, self-regulatory fatigue, mobile phone addiction, chain mediation model

## Abstract

**Background:**

Currently, the uncertainty and high pressure of the social environment, as well as the popularity of the Internet and mobile phones, have made mobile phone addiction a prevalent phenomenon among university students. This study explored the relationship between self-compassion and mobile phone addiction in university students and the mediating effects of negative attention bias and self-regulatory fatigue.

**Methods:**

The study used convenience sampling to recruit 800 Chinese university students to complete the Chinese version of the Self-Compassion Scale, the Negative Attention Bias Scale, the Self-Regulatory Fatigue Scale, and the Mobile Phone Addiction Scale. SPSS 24.0 and the Process 3.4 macro program were employed to generate the descriptive statistics and conduct the correlation analyses and mediating effect tests on the data.

**Results:**

The analyses revealed that self-compassion negatively predicted mobile phone addiction. Negative attention bias and self-regulatory fatigue individually mediated the effect of self-compassion on mobile phone addiction. Negative attention bias and self-regulatory fatigue also exhibited a chain mediation effect.

**Conclusions:**

The results of the study, from the perspective of self-compassion, have theoretical and practical implications for how to intervene in the mobile phone addiction of university students as well as to protect their physical and mental health.

## Background

A survey conducted by the China Internet Network Information Center in 2023 point out that there are 1.079 billion mobile Internet users in China, who spend an average of 29.1 hours a week online (1); university students are the main group of mobile Internet users (2). With smartphones and mobile Internet, university students can access information, socialize, and entertain themselves anytime, anywhere, and while this makes their lives easier and richer, it also brings a greater risk of mobile phone addiction (3). Addiction to mobile phones has been proven to significantly impact individuals’ physical and mental health, as well as their social relationships (4). This is especially serious for university students who are at critical stages of development. Additionally, the impact of COVID-19 on society as a whole has not yet been fully eliminated; although the economic situation is gradually recovering, university graduates continue to face new challenges, such as increased employment pressure (5). The number of 2024 national ordinary university graduates is expected to reach 11.79 million, an increase of 210,000 from 2023, while the unemployment rate of urban youths aged 16–24 years remained persistently high in the first half of last year (5). The dual pressures of academic and professional responsibilities exert a significant influence on the daily activities and interactions of university students, who rely heavily on mobile phones to navigate these demands (6). According to recent statistics, the global prevalence of mobile phone addiction is approximately 26.99% (7). A study carried out in China also found that 14.31% of university students were at a high risk of being addicted to mobile phones (2). University students are in a crucial phase of social adjustment and emotional growth, making them more susceptible to mental health issues and problematic behaviors (8). Therefore, reducing their mobile phone addictive behaviors and exploring the mechanisms behind them to promote their healthy development has become an issue of concern.

Reviewing past literature, we found that when individuals face external pressure and negative events, self-compassion will play a great protective role in their mental health (9). When an individual faces inadequacy, failure or pain, his or her ability to show compassion to himself or herself will have a positive impact on his or her academic achievement, physical behavior, psychological characteristics and other aspect. In the era just after COVID-19, self-compassion is an important protective factor for the physical and mental health of college students, helping college students improve their cognitive style (10), relieve negative emotions (11) supplement cognitive resources (12, 13) and take more adaptive actions to cope with external challenges (14). However, few studies in the past have linked the positive significance of self-compassion to college students’ mobile phone addiction, and the possible impact path behind it is still unclear. In summary, at a time when college students are facing many external challenges and pressures, exploring the impact of self-compassion on mobile phone addiction has practical significance, and provides practical guidance for improving the current situation of mobile phone addiction and dependence among college students. To address Based on these gaps, the present study attempts to investigate how self-compassion may reduce mobile phone addiction through negative attention bias and self-regulatory fatigue by integrating cognitive style (how individuals process information) and cognitive resource consumption (how individuals regulate effort and self-control), in order to provide theoretical support for how to effectively reduce mobile phone addiction.

Self-compassion is a psychological resource used to cope with difficulties and obstacles (15). In adversity, self-compassion helps people calm their negative emotions and adopt a non-judgmental attitude to reinterpret unpleasant events as meaningful or growth-promoting (9). Some studies have suggested that self-compassion can help university students develop a greater sense of prosperity and meaning in their lives (16). Conversely, those with low levels of self-compassion are more prone to engage in self-criticism when encountering stressful events (17). This can lead to more depressive moods and negative interpretive tendencies (18, 19) and, consequently, an avoidant attitude toward life (9). University students are vulnerable to stress (20) and self-compassion may be an important protective factor in improving their physical and mental health.

Mobile phone addiction refers to the phenomenon of addiction to excessive mobile phone use and the inability to control this usage, a behavior that can have a significant negative impact on individuals’ psychological and social functioning (21). With the rapid development of information technology, mobile phones have become essential mobile Internet terminals for university students (22), resulting in a high rate of addiction among them (23). Compensatory Internet use theory (CIUT) identifies a variety of motivations behind excessive or problematic technology use, all of which center on an individual’s response to adverse or stressful events (24). At present, universities not only have increasingly complex curricula but also have increasing requirements for students’ development in other aspects. Many students not only need to complete their daily courses and assignments and achieve high GPAs but are also expected to participate in various practical activities, such as academic programs and academic competitions. Additionally, to increase employment competitiveness, students must prepare for various qualification examinations or internships. As an effective coping strategy, self-compassion can help university students remain positive in stressful environments (25), have a stronger and more stable level of mental health (26), and escape avoidance and negative behaviors, which may help reduce the risk of mobile phone addiction (27). However, few studies have linked the positive significance of self-compassion to mobile phone addiction among university students, and the possible pathways behind its influence are unclear. As university students face many external challenges and pressures, it is of practical significance to explore the influence of self-compassion on mobile phone addiction and provide practical guidance for improving the current situation of mobile phone addiction and dependence prevalent among university students.

Therefore, we propose H1: Self-compassion negatively predicts mobile phone addiction among university students.

Negative attention bias is a cognitive style in which individuals have an attentional preference for negative information during information processing (28). Paying too much attention to negative information can increase the likelihood of mental health issues (29). The cognitive-behavioral model of pathological Internet use states that maladaptive cognition is the most critical and stable factor influencing the formation and maintenance of Internet addiction (30). A negative attention bias is defined as a tendency to focus on negative stimuli in one’s environment (31), leading to an increased attention to negative information and experiences (28). Responding to negative stimuli can lead individuals to experience increased negative emotions like anxiety (32). To alleviate their discomfort and stress, individuals indulge in playing mobile games or swiping on short videos to gain more instant gratification (33, 34); However, this may contribute to a vicious cycle, leading to excessive use of mobile phones in the long run (31). Self-compassion is effective in improving individuals’ coping and thinking styles when faced with negative information (15, 35). The mindfulness stress buffer hypothesis states that positive mindfulness, a key component of self-compassion, can improve cognitive appraisal and stress assessment by enabling individuals to process information with a balanced and calm demeanor, which, in turn, generates a distinctive buffer against the negative effects of stress (4, 36, 37). Individuals with high levels of self-compassion are more aware of their current emotions and perceptions (38), have reduced levels of negative attention bias, and develop a more objective understanding of life events (10).

Thus, we propose H2: Negative attention bias mediates the relationship between self-compassion and mobile phone addiction.

A state of continuous exhaustion, which is called self-regulatory fatigue, happens when a person consistently depletes their self-control resources to deal with external and internal negative factors (39, 40). According to the strength and resource theory of self-regulation, each individual’s capacity for self-control is limited (41, 42). This theory is particularly relevant for university students, who often find themselves in fast-paced and highly charged environments, which in turn can further deplete their already limited resources for self-control, making them more susceptible to a decrease in their power of self-control (43). Recent research adopting the I-PACE (Interaction of Person-Affect-Cognition-Execution) model has demonstrated that ego depletion, resulting from chronic self-control exertion, is a significant predictor of mobile phone addiction, emphasizing the mediating role of self-regulatory failure in the addictive process (44). Mobile phone addiction is the result of excessive self-comfort in individuals under the influence of negative factors (45) and is a type of self-avoidance for individuals facing negative events and negative emotions (46). A lack of self-control resources also increases the likelihood of problematic behaviors (47). However, there is a strong link between self-compassion and self-regulation (48). Self-compassion equips individuals with the necessary resources for self-regulation (12), while related intervention strategies significantly improve individuals’ self-control (48) and can help individuals reduce out-of-control behaviors, such as overeating, in a short time (49). Increased levels of self-compassion may restore inhibitory control that has been compromised by self-regulatory fatigue, which, in turn, may alleviate Internet or mobile phone addiction behaviors that accompany it (50, 51).

Accordingly, we propose H3: Self-regulatory fatigue mediates the relationship between self-compassion and mobile phone addiction.

The orienting attention/action readiness framework (OAAR) suggests that individual self-regulatory outcomes are determined by the interaction between action readiness and orienting attention (52). Negative attention bias can be defined as an individual’s inclination to evaluate, process, select, and remember negative information (53). This preference leads individuals to adopt more negative action readiness (54) and causes them to experience more negative emotions (55). Similarly, cognitive behavioral theory points to a person’s irrational perceptions of events as the root cause of negative emotions (56). Research has shown a strong correlation between self-regulatory fatigue and negative emotions (57). It intensifies the drain on people’s self-regulatory resources, when they attempt to change a negative emotion (42, 58). As a result, they are more likely to succumb to self-regulatory fatigue. This state of fatigue often leads individuals to ignore long-term benefits and pursue immediate gratification. According to the OAAR framework, addictive cell phone behavior among university students may also be problematic because negative attention bias aggravates self-regulation (52).

Hence, we propose H4: Negative attention bias and self-regulatory fatigue jointly play a chain-mediating role in the relationship between self-compassion and mobile phone addiction.

**Figure 1:**
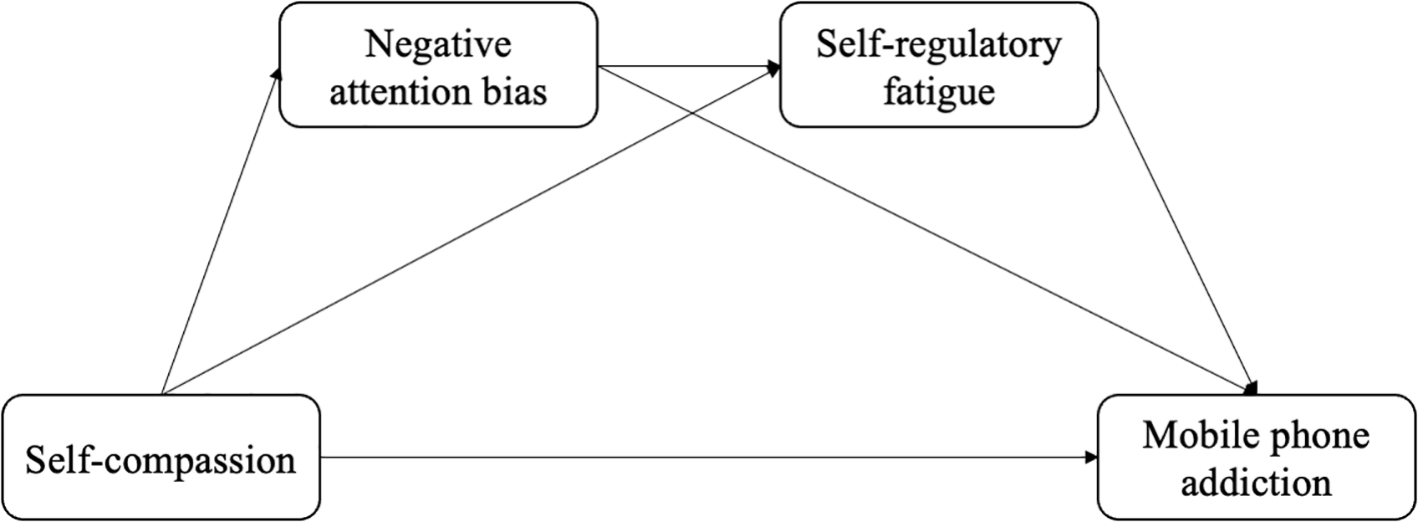
Proposed model.

In summary, this study attempts to examine the relationship between self-compassion and mobile phone addiction and to construct a chain mediation model (see **Figure 1**).

## Method

### Participants

To recruit university students from Guangdong Province, China, convenience sampling was employed. A total of 917 questionnaires were distributed via an online platform, out of which 117 were excluded due to invalid responses (such as lack of seriousness, incomplete answers, or patterned responses). Finally, 800 valid questionnaires were obtained, yielding a validity rate of 87.2%. Of these participants, 280 (35.0%) were male and 520 (65.0%) were female, with ages ranging from 18 to 28 years (M = 19.09; SD = 1.21). The study received approval from the ethics committee of the institution where it was conducted.

### Tools

#### Self-compassion scale

The Self-Compassion Scale developed by Neff (59) and revised by Chen, Yan (60) was used to assess the level of self-compassion among university students. The scale comprises 26 items, such as ‘I am dissatisfied and critical of my own shortcomings and deficiencies’ and ‘I try to face painful things with a calm mind when they happen’. The items are divided across six dimensions: kind-to-self, self-criticism, common humanity, self-isolation, stillness in the present moment, and over-indulgence. All items are scored on a 5-point Likert scale ranging from 1 (‘never’) to 5 (‘always’). Higher total scores indicate higher levels of self-compassion. The scale is applicable to the Chinese population and has good reliability (60). In this study, the Cronbach’s alpha coefficient of the scale was 0.89, and the Cronbach’s alpha coefficients for the six dimensions were 0.86 for kind-to-self, 0.78 for self-criticism; 0.77 for common humanity, 0.83 for self-isolation, 0.85 for stillness in the present moment, and 0.73 for over-indulgence.

#### Negative attention bias scale

The negative subscale of the Positive and Negative Information Attention Bias Scale, developed by Dai, Li (61) was used to assess negative attention bias. The subscale comprises 10 items, such as ‘I can’t forget the times when I did poorly on something’ and ‘I always pay attention to past scenarios that made me feel bad’. Items are scored on a 5-point Likert scale ranging from 1 (‘not at all consistent’) to 5 (‘completely consistent’). Higher total scores indicate higher negative attention bias. The scale is applicable to the Chinese population and has good reliability (61). The Cronbach’s alpha coefficient in this study was 0.92.

#### Self-regulatory fatigue scale

The Self-Regulatory Fatigue Scale developed by Nes et al. and revised by Wang et al. (62, 63) was used to assess participants’ levels of self-regulatory fatigue. It comprises 16 items, such as ‘I feel energized’, ‘I have a hard time carrying out my exercise program’, and ‘Staying in touch with friends is easy for me’. The items are divided across cognitive, emotional, and behavioral subscales and are rated on a 5-point Likert scale ranging from 1 (‘strongly disagree’) to 5 (‘strongly agree’). Higher total scores indicate greater self-regulatory fatigue. Questions 1, 2, 5, 9, and 14 are reverse-scored. The scale is applicable to the Chinese population and has good reliability (63). Cronbach’s alpha coefficient for the scale in this study was 0.87, and the alpha coefficients for the subscales were 0.74 for cognitive, 0.69 for emotional, and 0.75 for behavioral.

#### Mobile phone addiction scale

The Mobile Phone Addiction Scale developed by Xiong, Zhou (64) was used to assess the degree of mobile phone addiction among participants. The scale comprises 16 items, such as ‘I prefer cell phone chats to direct face-to-face communication’, ‘I can’t concentrate in class because of phone calls or text messages’, and ‘I am often afraid that my mobile phone will turn off automatically’. Items are scored on a 5-point Likert scale ranging from 1 (‘not at all consistent’) to 5 (‘completely consistent’). The scale comprises four dimensions: withdrawal symptoms (negative physiological or psychological reactions to not engaging in cell phone activities), salient behaviors (mobile phone use occupies the center of thinking and behavioral activities), social comfort (the role of mobile phone use in interpersonal interactions), and mood changes (emotional changes caused by mobile phones), with higher scores representing more severe cell phone addiction. The scale is applicable to the Chinese population and has good reliability (64). The Cronbach’s alpha coefficient of the scale in this study was 0.92, and the alpha coefficients for the four dimensions were 0.83 for withdrawal symptoms, 0.79 for salient behaviors, 0.83 for social comfort, and 0.67 for mood changes.

### Data analysis

The data were analyzed using SPSS 24.0 and the Process 3.4 macro to perform descriptive statistics, correlation analyses, and tests for chain mediation effects.

## Results

Common method bias was tested using Harman’s one-way test (65), because the data were self-reported by the participants. The analysis revealed 11 common factors with eigenvalues greater than 1, extracted without rotation. The first factor accounted for 24.89% of the variance, significantly below the 40% threshold, suggesting that serious common method bias was not present.

**Table 1** shows the descriptive statistics and correlation analysis of variables. All study variables were significantly correlated. The results of the independent samples t-test showed significant differences in the levels of self-compassion (t = −2.03, *p* = 0.021) and self-regulatory fatigue (t = −1.79, *p* = 0.037) among participants of different sexes. The results of the one-way analysis of variance showed a significant difference in negative attention bias (F(8, 791) = 2.76, *p* = 0.005) among participants of different ages. Home location (urban, rural, and township) significantly affected self-regulatory fatigue (F(2, 797) = 3.52, *p* = 0.030) and negative attention bias (F(2, 797) = 3.77, *p* = 0.023). Additionally, mothers’ education level significantly impacted negative attention bias (F(4, 795) = 3.42, *p* = 0.009).

**Table 1:** Descriptive statistics and correlation analysis of variables.

Variable	*M*	*SD*	1	2	3
1 Self-compassion	83.64	12.45	–		
2 Negative attention bias	30.22	7.75	-0.49^***^	–	
3 Self-regulatory fatigue	42.97	9.20	-0.70^***^	0.53^***^	–
4 Mobile phone addiction	43.63	11.19	-0.45^***^	0.45^***^	0.52^***^

N=800, ^***^ *p*<0.001

By contrast, father’s education level and family economic status did not show significant associations with any of the study variables. Based on these findings, we included sex, age, home location, and mother’s education level as control variables in the mediation model analysis using the PROCESS macro.

**Figure 2:**
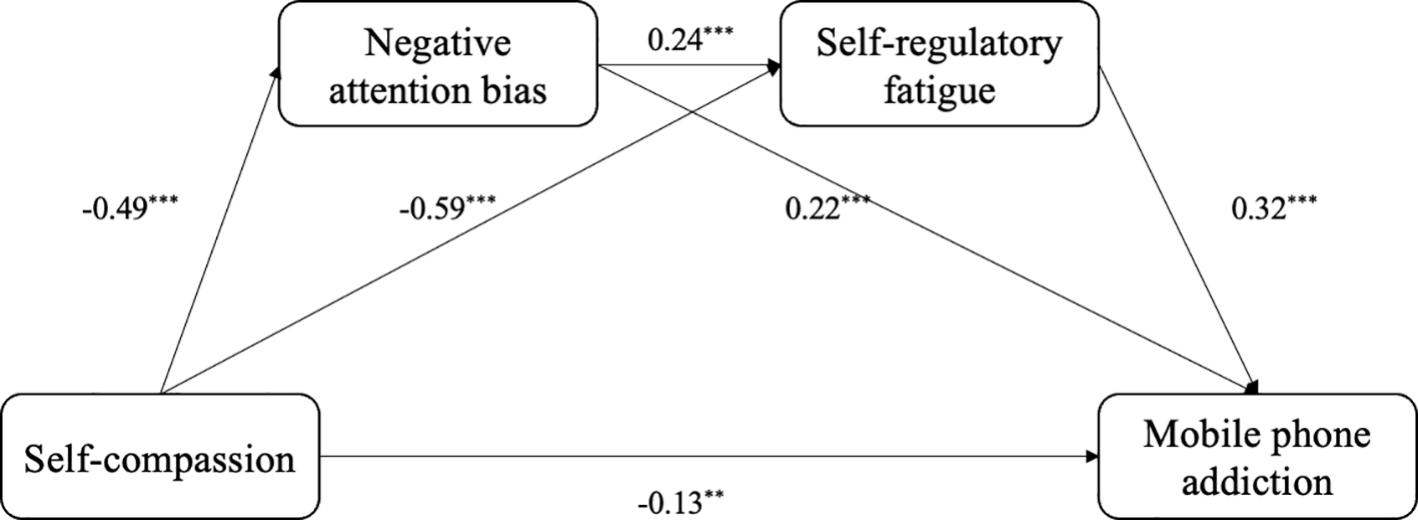
Chain mediation model. **p<0.01, ***p<0.001.

Model 6 in the Process macro program developed by (66) was utilized to examine the chain mediation model(see **Table 2**, **Figure 2**). The results indicated that self-compassion significantly and negatively predicted mobile phone addiction(β = −0.46, *p* < 0.001). When negative attention bias and self-regulatory fatigue were introduced as mediators, self-compassion remained significant in predicting mobile phone addiction (β = −0.13, *p* = 0.026), self-compassion significantly predicted negative attention bias (β = −0.49, *p* < 0.001) and self-regulatory fatigue (β = −0.59, *p* < 0.001), and negative attention bias significantly predicted self-regulatory fatigue (β = 0.24, *p* < 0.001). Furthermore, both negative attention bias (β = 0.22, *p* < 0.001) and self-regulatory fatigue (β = 0.32, *p* < 0.001) reached significant levels of predictive power for mobile phone addiction.

**Table 2:** Chain mediation model test.

Regression equation		Overall fit indices			Significance of the regression coefficients		
Outcome variables	Predictors	*R*	*R^2^*	*F*	*β*	*95%CI*	*t*
Mobile phone addiction	Self-compassion	0.46	0.21	43.20^***^	-0.46	[-0.52,-0.40]	-14.49^***^
Negative attention bias	Self-compassion	0.50	0.25	54.22^***^	-0.49	[-0.55,-0.42]	-15.75^***^
Self-regulatory fatigue	Self-compassion	0.74	0.55	159.46^***^	-0.59	[-0.64,-0.53]	-21.24^***^
	Negative attention bias				0.24	[0.19,0.30]	8.70^***^
Mobile phone addiction	Self-compassion	0.57	0.33	54.71^***^	-0.13	[-0.21,-0.04]	-3.02^**^
	Negative attention bias				0.22	[0.15,0.29]	6.24^***^
	Self-regulatory fatigue				0.32	[0.23,0.40]	7.37^***^

^**^ *p*<0.01,^***^ *p*<0.001. Gender, age, home location and mother education level were done as control variables included in the model. The study variables were standardized.

The results of the mediating role test showed (see **Table 3**) that bootstrap 95% confidence intervals did not include zero, indicating significant mediation effects. Specifically, negative attention bias mediated the effect of self-compassion on mobile phone addiction, with a mediating effect of −0.11, which accounted for 23.91% of the total effect; thus, H2 was supported. Self-regulatory fatigue mediated the effect of self-compassion on mobile phone addiction, with a mediating effect of −0.19, accounting for 41.30% of the total effect; thus, H3 was supported. The chain mediation effect of negative attention bias and self-regulation fatigue also reached a significant level with a mediation effect of −0.04, representing 8.70% of the total effect, thereby supporting H4.

**Table 3:** Chain mediation model effect size analysis.

Effect Type	*Effect*	*SE*	*95%CI*
Total effect	-0.46	0.03	[-0.40, -0.46]
Direct effect	-0.13	0.04	[-0.21, -0.04]
Indirect effect of negative attention bias	-0.11	0.02	[-0.15, -0.07]
Indirect effect of self-regulatory fatigue	-0.19	0.03	[-0.25, -0.13]
Indirect effect of negative attention bias and self-regulatory fatigue	-0.04	0.01	[-0.06, -0.02]

Effect size. SE and 95% CI refer to the standard errors, lower and upper 95% confidence intervals of the indirect effects estimated by the bias-corrected percentile Bootstrap method, respectively.

## Discussion

Along with the rapid development and widespread popularization of information technology, mobile Internet and smartphones have become an indispensable part of university students’ daily study, work, and life. However, prolonged mobile phone usage increases the risk of addiction. University students are in a transitional stage of emotional development and social adaptation, which makes them more susceptible to problematic behaviors like mobile phone addiction. This addiction can have a relatively greater negative impact on their physical and mental health (3). Additionally, the external environment in recent years may have increased the risk of mobile phone addiction among university students. Since the COVID-19 outbreak, the economy, education, and life in China and worldwide have been significantly impacted and, to this day, some of the effects have not been entirely eliminated (5). Against this background, university students face unprecedented challenges in various aspects, such as schooling and employment, and these external pressures may push them to turn to the virtual world of mobile phones to escape the reality of life (6). Therefore, to protect the physical and mental health of university students, it is necessary to focus on ways to reduce mobile phone addiction. This study examined the effect of self-compassion on mobile phone addiction by constructing a chain mediation model from the perspective of cognitive styles and cognitive resources and exploring the underlying mechanisms of negative attention bias and self-regulation fatigue based on self-compassion. This psycho-protective factor can help individuals maintain a positive attitude in the face of negative events. The study results demonstrated that self-compassion in university students could predict their mobile phone addiction behaviors, both directly and indirectly. Additionally, this study’s findings, from the perspective of self-compassion, offer theoretical and practical guidance on intervention strategies for mobile phone addiction among university students, contributing to the protection of their physical and mental health.

This study established that self-compassion significantly predicted mobile phone addiction; that is, the higher an individual’s level of self-compassion, the less addictive the mobile phone behavior, which is consistent with previous research findings (27, 67). Self-compassion is a psychological conditioning resource that individuals can adopt in the face of external stress or negative events (15). Individuals with high levels of self-compassion take effective actions to adjust their emotions and cope with adverse events. CIUT suggests that the core of excessive cell phone use comes from an individual’s adverse reactions to external events (24). At present, university students are facing increasing academic and employment requirements. The pressure generated by external factors can prevent individuals from satisfying their basic psychological needs, and individuals may generate defense mechanisms to seek ways to satisfy their needs. The strong emotions and fast updating of information stimulated by cell phones can help individuals obtain temporary psychological compensation, but also strengthen the process of cell phone use (67). Individuals with lower levels of self-compassion have weaker psychological functioning, and their basic psychological needs are more likely to be impaired. Therefore, they are more likely to choose cell phones to escape the pressure of reality. However, individuals with high self-compassion tend to adopt more appropriate ways to adjust to negative emotions, prompting them to convert into more positive psychological traits, thus avoiding the emergence of mobile phone addictive behaviors.

The results also suggest that negative attention bias plays a mediating role in the relationship between self-compassion and mobile phone addiction. In other words, self-compassion can not only directly affect university students’ addictive behavior towards mobile phones, but it can also influence this behavior through negative attention bias. This lends further support to the cognitive-behavioral model of pathological internet use, which posits that the most critical factor leading to the formation of internet addiction is the way individuals perceive external information (30). University students with negative attention bias are more likely to focus on negative stimuli in the information they receive from the outside world, resulting in more negative emotions and undesirable behavioral tendencies (30). This group has a greater need to relieve stress and improve their mood and is more likely to be attracted by the instant gratification brought about by cell phones, thus developing mobile phone addiction behaviors (31, 34). On the other hand, the results validate the impact of self-compassion on mobile phone addictive behaviors by improving cognitive styles. A key aspect of self-compassion is a balanced and non-judgmental cognitive style, which enables university students to process external information more objectively, thus making accurate cognitive and stress assessments (36). Therefore, self-compassion can help university students recognize their own emotions and judge their situations calmly, thus reducing negative attention bias and adopting more appropriate methods to solve the difficulties they presently face (10, 38).

Likewise, this study validated that self-compassion can predict university students’ mobile phone addiction through the mediating role of self-regulatory fatigue, a result consistent with the power and resource theories of self-regulation (41). The results also verified the influence of self-compassion on university students’ mobile phone addictive behavior by supplementing their cognitive resources and changing their cognitive styles. When individuals are faced with stressful events and negative emotions, they use up their limited cognitive resources to manage these situations. However, when these resources are excessively consumed, it leads to a state known as regulatory fatigue. This state greatly impacts self-control related activities, resulting in problematic behaviors like addiction (41). University students are in a critical period of transition from student to adult status, facing new changes and challenges, such as in academics, employment, and life, while lacking experience in coping with and dealing with negative events (43).

Consequently, when faced with similar stressful events, this group develops more negative emotions, requires more cognitive resources for regulation, and is more likely to experience physical and mental exhaustion (43). As a form of solace, cell phones often allow people to escape the reality of their emotions when they are unable to self-regulate (45, 46). However, self-compassion can help individuals regain self-control and thus reduce mobile phone addictive behaviors (12). When the level of self-compassion of university students increases, on the one hand, they can view the existence of negative emotions more rationally; on the other hand, they may have sufficient cognitive resources to deal with their emotions and solve the difficulties they encounter directly, rather than using cell phones as escapes.

Moreover, the results showed that self-compassion affects negative attention bias, and a higher negative attention bias triggers self-regulatory fatigue, which, in turn, contributes to mobile phone addiction. Self-compassion contributes to the improvement of cognitive styles and the supplementation of cognitive resources, whereas cognitive styles are closely related to self-regulation. According to the orienting attention and action readiness framework, the outcome of self-regulation is influenced by directed attention and action readiness (52). In today’s information society, university students, who are the primary users of the Internet, are subjected to vast amounts of external information (68). Individuals are more likely to experience negative emotions and tend to exhibit avoidant behavior when they have a preference for documenting and receiving negative content in their messages (54, 55). They need to utilize more cognitive resources for self-control, to manage these negative emotions and prepare for action, which subsequently makes them more susceptible to self-regulatory fatigue (58). Therefore, while negative attention bias exacerbates university students’ negative experiences in the face of external stressors and increases the cognitive burden of self-regulation, which, in turn, increases the risk of mobile phone addiction after a failure of regulation, self-compassion helps students cope with internal and external negativity and reduces their susceptibility to stress, thus reducing their problematic behaviors (50, 51). Notably, although the chain-mediated effect appears modest in magnitude, it offers meaningful theoretical and practical insights. Specifically, this sequential pathway highlights how self-compassion reduces mobile phone addiction through both a shift in cognitive processing (i.e., reduced negative attention bias) and conservation of cognitive resources (i.e., lower self-regulatory fatigue). This dual-path explanation enriches the existing literature by integrating cognitive and resource-based mechanisms, offering a more comprehensive understanding of how psychological traits like self-compassion influence behavioral outcomes. Furthermore, given the increasing maturity of intervention programs aimed at enhancing self-compassion (e.g., compassion-focused therapy, mindfulness-based training) (69, 70), our findings provide a foundation for practical applications in reducing mobile phone addiction through targeted psychological support in educational and clinical settings.

Given the versatility and usefulness of smartphones, their annual use is continually increasing (71). Simultaneously, however, there has been a major shift in people’s lives and a global decline in life satisfaction due to the COVID-19 pandemic and its global aftermath (72). People are facing more external pressures, and mental health problems have increased among the younger population, represented by university students (73). University students may turn to smartphones, a popular technological device, to cope with their adverse psychological state; however, this increases their screen time and the risk of smartphone addiction (74). To break this cycle, this study confirmed the influence of self-compassion on university students’ mobile phone addictive behaviors, revealed the intrinsic mechanism between negative attention bias and self-regulatory fatigue, and is of positive significance for understanding the causes of mobile phone addictive behaviors and interventions for university students facing external pressures. Additionally, this study introduces the protective factors of self-compassion. It emphasizes the value of individual psychological traits in safeguarding physical and mental health from the perspective of improving cognitive styles and supplementing cognitive resources. The results also provide insights into how to help university students establish healthier mobile phone usage habits, thereby preventing or alleviating cell phone addiction. Schools can not only shift students’ attention from cell phones to academics by appropriately adjusting their teaching methods and increasing their interest in learning, but also promote the development of positive psychological resources and emotional regulation by providing mental health education, workshops, and lectures that help students improve their level of self-compassion or self-regulation. At the family level, parents can encourage their children to adopt a self-compassionate attitude in the face of difficulties and failures by understanding and supporting their children’s emotional needs, which can help enhance the emotional stability of university students and reduce mobile phone addiction caused by emotional stress.

Despite the valuable insights gained from this study, several limitations should be noted. First, the data were collected using self-reported questionnaires, which may introduce subjective bias and affect the reliability of the results. Future research could combine self-report measures with more objective evaluation methods (e.g., behavioral tracking, peer reports) to improve data accuracy. Additionally, the study relied on convenience sampling from a single Chinese province (Guangdong). This limits the external validity and generalizability of the findings, as university students in different regions or cultural settings may exhibit varying levels of self-compassion, cognitive biases, and mobile phone usage patterns. Future research should aim to include more diverse and representative geographic and demographic samples, such as more provinces, ethnic groups, or data from different countries, to enhance generalizability. Second, the cross-sectional design of this study limits the ability to draw causal inferences between self-compassion, negative attention bias, self-regulatory fatigue, and mobile phone addiction. Although the proposed model is theoretically grounded, the directionality of the relationships cannot be definitively established. To confirm the temporal and causal relationships, future research should employ longitudinal designs to track changes over time, or experimental studies that manipulate self-compassion (e.g., through interventions or training programs) to observe their effects on cognitive style, self-regulation, and behavioral outcomes. Such designs would provide stronger empirical support for the proposed mediating mechanisms and contribute to a more robust theoretical model. Third, focusing on the era in which the effects of the pandemic have not yet been eliminated, and university students face difficulties in furthering their education and employment, this study examined the mechanism of self-compassion’s influence on mobile phone addictive behaviors from two perspectives: cognitive modality and cognitive resources. However, with the development of the economic situation, it is necessary to further explore whether the model of this study changes in other future contexts and whether the mechanism of self-compassion’s influence on an individual’s mobile phone addiction will change. Fourthly, despite the fact that China’s national economy has gradually shown a sustained and stable recovery since the outbreak of the pandemic, the deep-rooted impact on employment is still ongoing, with an increasing number of university graduates facing grim employment situations, employment pressure, and other problems, as well as the accompanying difficulties of internships and pressure to further their studies (5). The unique social contexts encountered and characterized by Chinese university students may not be the same as those experienced in other countries during the post-pandemic recovery period. This could lead to differences in the cognitive approaches and resources used for managing cell phone usage under stress between individuals in China and those in Western countries. Future research should therefore explore this research model in a cross-cultural context. Lastly, although Harman’s one-factor test was used in this study to test for common method variance, we recognize that this approach has limitations and may not fully eliminate concerns regarding method bias. Future research is encouraged to adopt more rigorous techniques, such as confirmatory factor analysis (CFA) with a marker variable or a latent method factor, to provide a more comprehensive assessment of potential common method variance.

## Conclusion

In this study, self-compassion negatively predicted mobile phone addictive behavior. Negative attention bias and self-regulatory behaviors individually mediated the effect of self-compassion on mobile phone addictive behaviors. Negative attention bias and self-regulatory fatigue also exhibited a chain-mediation effect on the relationship between self-compassion and mobile phone addiction.

## Data Availability

The raw data supporting the conclusions of this article will be made available by the authors, without undue reservation.
